# Migration of an intrauterine device to the posterior urethra with stone formation: a case report

**DOI:** 10.3389/fmed.2024.1449443

**Published:** 2024-08-22

**Authors:** Chuanfeng Liu, Yongqiang Xia, Qingtan Pang, Zichao Zhao, Jianfang Zhao

**Affiliations:** ^1^Department of Urology, Linyi Maternity and Child Health Care Hospital, Linyi, Shandong, China; ^2^Department of Gynecology, Linyi Maternity and Child Health Care Hospital, Linyi, Shandong, China

**Keywords:** intrauterine device, migration, urethra, bladder stone, endoscopy

## Abstract

Migration of an intrauterine device (IUD) to the posterior urethra with stone formation has not been previously reported in the literature. A 42-year-old female patient presented to the gynecology clinic with a complaint of “discovered vaginal mass for 2 years, with growth for 5 days.” She was referred to urology on suspicion of IUD migration to the bladder. Physical examination revealed a hard mass palpable on the anterior vaginal wall. Laboratory tests showed normal blood counts, and urinalysis indicated a mild urinary tract infection. Ultrasound and pelvic X-ray indicated IUD migration to the bladder and bladder stones. Cystoscopy revealed that the IUD had migrated to the posterior urethra with stone formation. Holmium laser was used to fragment the stones encasing the IUD’s one arm, and the IUD was successfully removed with grasping forceps. The patient had a urinary catheter placed for 10 days and was followed up for 20 days. During the follow-up, there were no lower urinary tract symptoms (LUTS) or vaginal leakage. To our knowledge, we report the first case of an IUD migrating through the vesicovaginal space to the posterior urethra. Endoscopic removal of the IUD is feasible and safe. Urologists and gynecologists should not limit their diagnosis to IUD migration to the bladder but should also consider the possibility of urethral migration.

## Introduction

The intrauterine device (IUD) is recognized globally as a widely accepted contraceptive method for women of reproductive age due to its simplicity, safety, efficacy, and reversibility. In East Asia and Southeast Asia, the IUD is the most commonly used contraceptive method (1). In China, the number of women using IUDs for contraception is greater than the total number in the rest of the world combined (2). However, some common complications may occur within the first few months after IUD insertion, such as pelvic pain and abnormal bleeding. IUD migration is a rare but serious complication, with the device primarily migrating to the abdominal cavity and less frequently to visceral organs (ovaries, adnexa, rectum, sigmoid colon, appendix, bladder), iliac vessels, or subcutaneous tissue (3). Since uterine perforation is asymptomatic in most cases (4), the symptoms experienced by patients vary depending on the specific tissue or organ to which the IUD has migrated. The diagnosis of IUD migration primarily relies on imaging studies. The main treatment options for removing a migrated IUD include endoscopy, minimally invasive laparoscopic surgery, and open surgery.

Reports also indicate that the bladder is the most common site for IUD migration (5). The incidence of bladder wall penetration is between 0.05 and 1.3 per 1,000 women; over time, it is likely to result in bladder stone formation (6). Since most experience treating IUD migration comes from case reports, these cases are often difficult to manage. We report a case initially misdiagnosed as IUD migration to the bladder before cystoscopy, where intraoperatively, it was found that one of the IUD’s arms had perforated into the posterior urethra with stone formation. The IUD was successfully removed using forceps during cystoscopy. To our knowledge, this is the first report of an IUD perforating into the posterior urethra, and it is presented as follows.

## Case description

**Figure 1:**
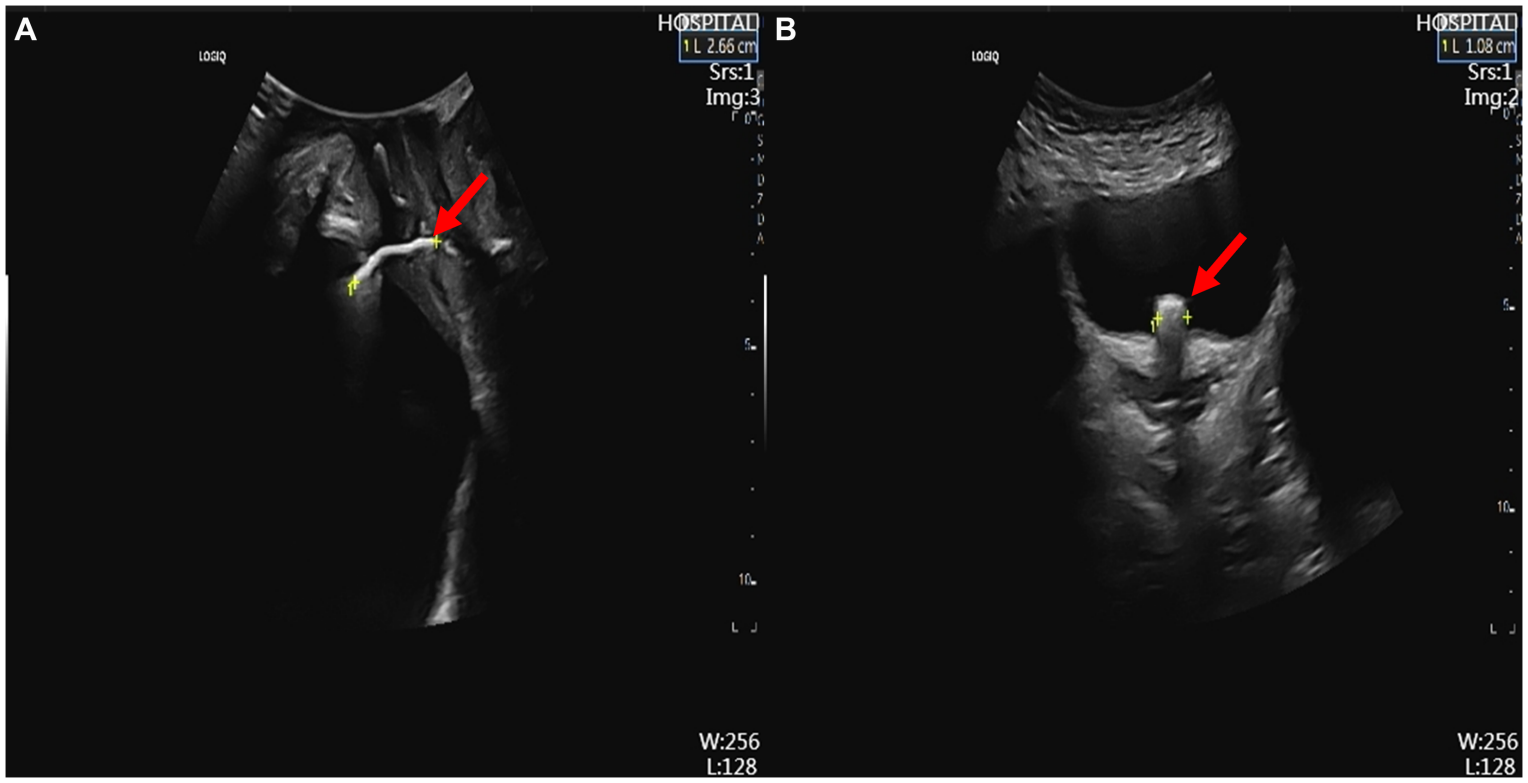
Ultrasound findings. **(A)** Transperineal ultrasound showing the IUD in the vesicovaginal space (red arrow). **(B)** Abdominal ultrasound showing one arm of the IUD penetrating the bladder wall, indicating bladder stone formation (red arrow). IUD, intrauterine device.

**Figure 2:**
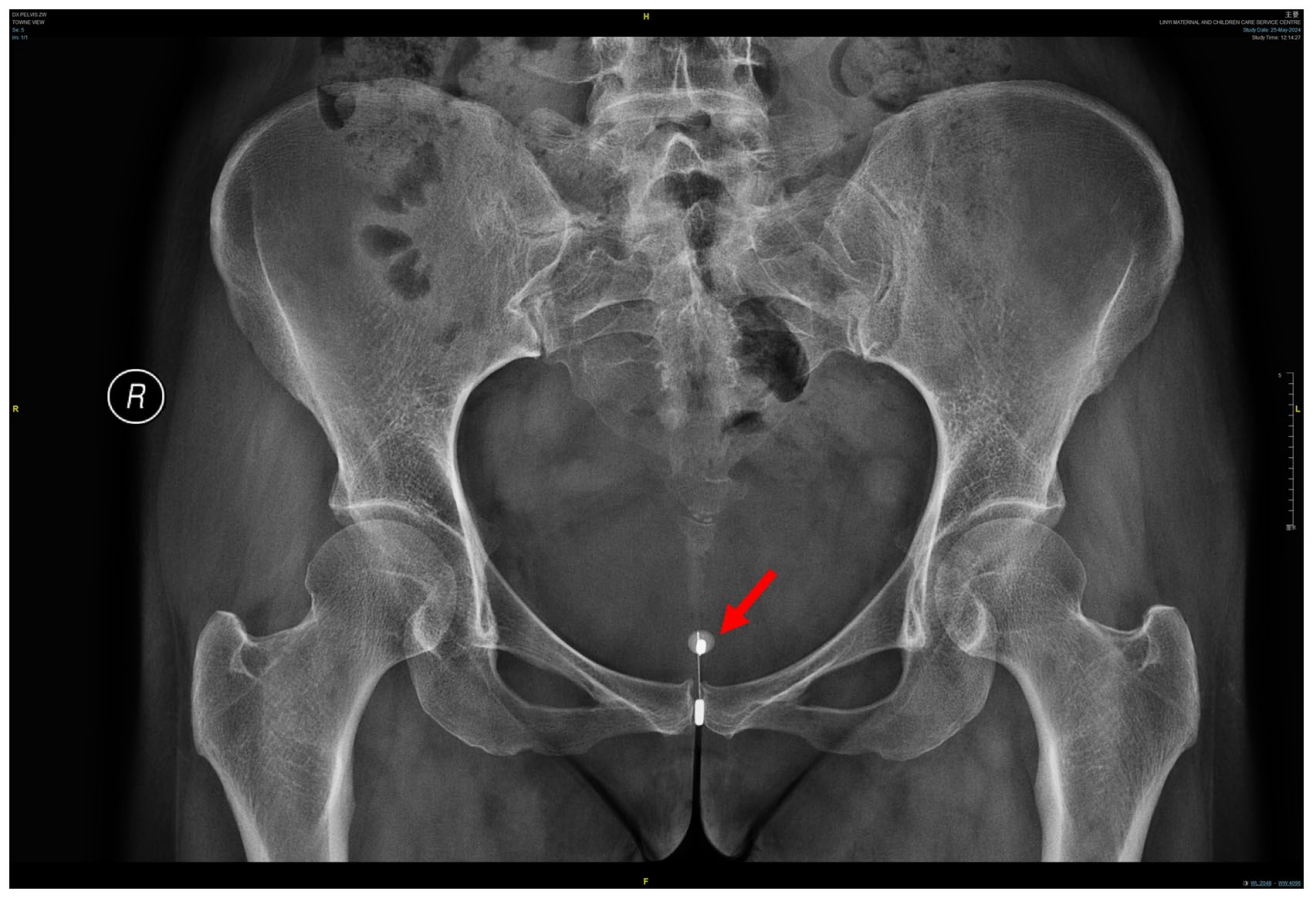
Pelvic X-ray showing the IUD, with a round, high-density shadow around one arm of the IUD, indicating bladder stone formation (red arrow). IUD, intrauterine device.

The patient is a 42-year-old woman with a BMI of 33.3 kg/m^2^, presenting with a chief complaint of “discovered vaginal mass for 2 years, with growth for 5 days.” She was referred to our department after an outpatient examination suspected IUD migration to the bladder. Sixteen years ago, 50 days postpartum, she had an IUD inserted at a primary health center. After insertion, she experienced mild vaginal bleeding, which gradually resolved, and she did not undergo regular follow-up. Fifteen years ago, she underwent a suction curettage abortion under ultrasound guidance due to pregnancy. The doctor and the patient believed that the IUD had expelled itself, so another IUD was inserted. Eleven years ago, the IUD was removed, and seven years ago, she became pregnant and delivered one child vaginally. Two years ago, she noticed a mass in the vaginal wall while bathing, which was asymptomatic and was not evaluated. One month ago, she experienced dysuria and hematuria after sexual intercourse, suspected to be a “urinary tract infection,” which resolved after taking antibiotics. Five days ago, while bathing, she noticed that the vaginal wall mass had increased in size. Physical examination: In the lithotomy position, with the assistance of a vaginal speculum, a black metal foreign body was visible approximately 3 cm from the hymenal rim on the anterior vaginal wall, not protruding from the mucosal surface; it was hard on palpation and non-tender. Urinalysis: Urine leukocyte esterase +−, red blood cells 0.0/μL, white blood cells 15.0/μL. Transperineal and abdominal ultrasound: The IUD was visible in the vesicovaginal space, with one arm penetrating the vaginal wall and the other penetrating the bladder wall with bladder stone formation (Figure 1). Pelvic anteroposterior X-ray: The IUD was visible in the pelvic cavity (Figure 2). Preoperative diagnosis: IUD migration to the bladder with bladder stone formation. We proceeded with cystoscopy for further evaluation.

**Figure 3:**
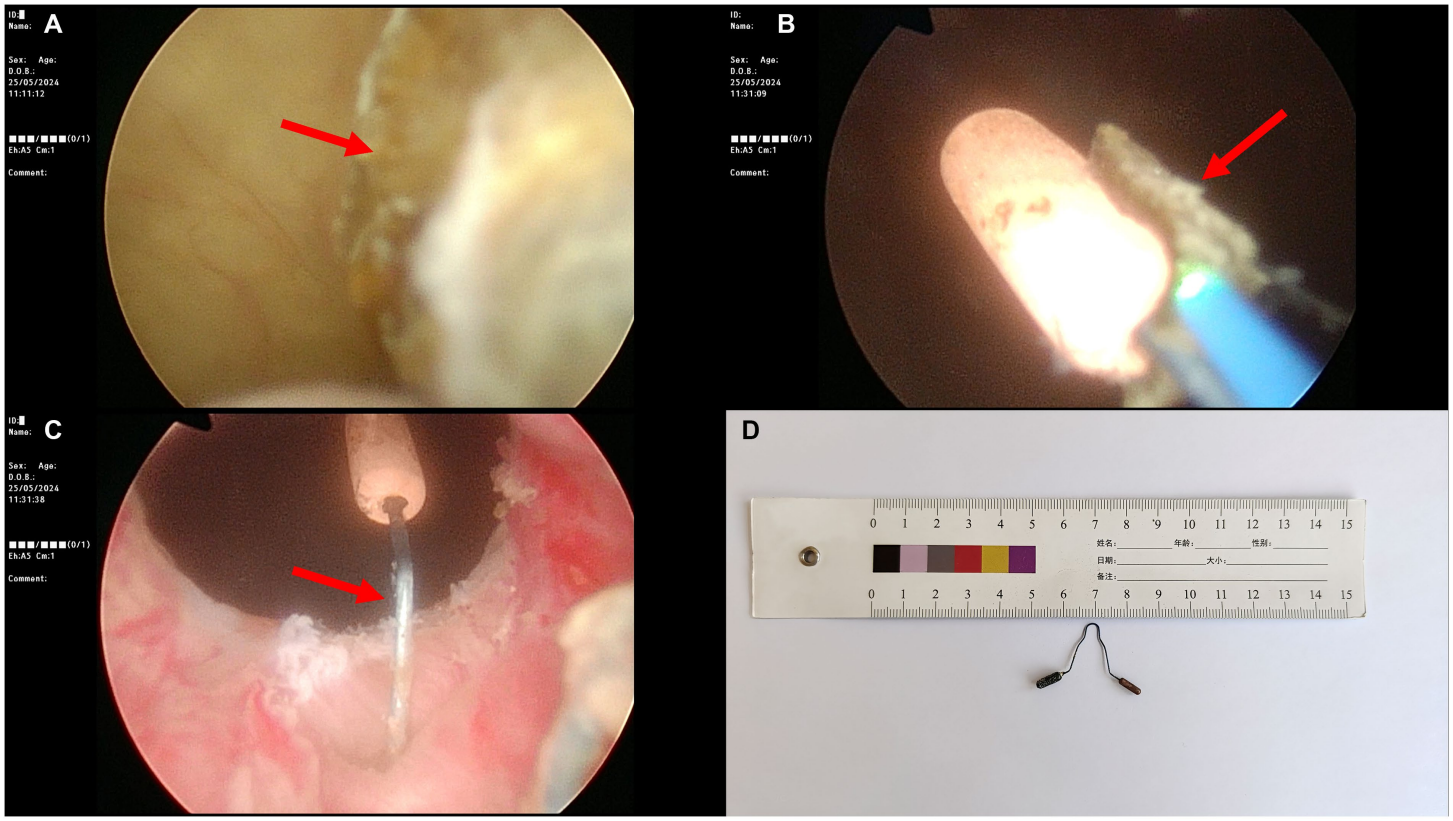
Cystoscopic findings. **(A)** Endoscopic view showing bladder stone protruding into the bladder from the urethral orifice (red arrow). **(B)** A holmium laser is used to fragment the stone, encasing the IUD’s one arm (red arrow). **(C)** One arm of the IUD perforating into the bladder at the 5 o’clock position of the posterior urethra (red arrow). **(D)** Retrieved “V”-shaped IUD. IUD, intrauterine device.

The patient was placed in the lithotomy position, and local anesthesia was administered to the urethral mucosa. Cystoscopy revealed a yellow-brown stone approximately 1.0 cm in length protruding into the bladder from the urethral orifice, which did not move upon irrigation. The stone was fragmented using a holmium laser (1.6 J, 30 Hz), revealing the IUD’s one arm in the center of the stone. The arm perforated into the bladder at the 5 o’clock in the posterior urethra (Figure 3). The tail end of the IUD was grasped with forceps, and the IUD was completely removed from the body. A 16Fr double-lumen catheter was placed to prevent the formation of a fistula between the urethra and vagina. The total operation time was 40 min.

The catheter was removed 10 days postoperatively, and at the 20-day follow-up, there were no lower urinary tract symptoms or vaginal leakage.

## Discussion

Although the incidence of IUD migration is low, it is a clinical issue worthy of attention due to the large user population (159 million women of reproductive age worldwide use this contraceptive method) and the potential for serious complications (1). The prerequisite for IUD migration is uterine perforation, with large studies reporting a uterine perforation rate of 0.4–2.2 per 1,000 women (4). Due to the asymptomatic nature of perforations, the actual rate may be higher. Uterine perforation can be classified as partial or complete. Partial perforation can progress to complete perforation, leading to migration. Although IUD migration into the urinary system is rare, there have been reports in the literature of migration to the bladder, ureters, and urethral orifice (6–8). Given the anatomical proximity of the bladder and uterus, it is unsurprising that the bladder is a common site for IUD migration within the urinary system.

The uterine wall heals rapidly, making it difficult to identify scar tissue via laparoscopy a few days or weeks after perforation (9). Therefore, simple uterine perforations are often asymptomatic. The symptoms experienced by patients vary depending on the organ or tissue to which the IUD has migrated. When intestinal perforation occurs, the main symptoms include fever, abdominal pain, hematochezia, and intermittent diarrhea (10, 11). When perforation occurs in the lower urinary tract, symptoms may include difficulty urinating, frequent urination, suprapubic pain, hematuria, and recurrent urinary tract infections (4). When the ovary is perforated, it can cause pelvic pain and dyspareunia (12). Notably, in this patient, the IUD was partially exposed in the vagina, presenting only with the chief complaint of a “vaginal mass” and no history of recurrent urinary tract infections. This could be due to minimal irritation caused by the IUD’s side arm penetrating the bladder. The IUD migrated one year after insertion when the patient experienced an unintended pregnancy. However, due to the asymptomatic nature of the migration, local doctors attributed it to IUD expulsion, delaying the diagnosis of migration by 15 years.

The mechanisms of uterine perforation are not fully understood and can be broadly categorized into two types: immediate perforation at the time of insertion and chronic secondary perforation (4). Reported risk factors for uterine perforation include breastfeeding, less than six months postpartum, low parity, high abortion rates, inflammation associated with IUD use, uterine contractions caused by delivery or sexual activity, peritoneal fluid movement, intestinal peristalsis, and lack of physician experience (4, 6, 13, 14). The migration of an IUD to the posterior urethra with its main body embedded in the vesicovaginal space has not been reported before, which piqued our interest. In this case, the patient became pregnant one year after IUD insertion, suggesting early migration likely due to immediate perforation during the insertion procedure. Vaginal bleeding after IUD insertion further supports our hypothesis. Seven years ago, the patient delivered vaginally with the IUD already migrated. Intestinal peristalsis, peritoneal fluid movement, increased abdominal pressure, and compression from the gestational sac likely caused the IUD to migrate through the bladder’s uterine recess to the vesicovaginal space, eventually perforating into the vagina and posterior urethra due to chronic inflammation associated with the IUD. However, given the patient’s history of curettage, another plausible hypothesis is that the IUD migrated one year after insertion. The patient underwent suction curettage at a local health center, where the physician’s lack of caution and subsequent pregnancy may have caused the IUD to migrate to the posterior urethra. In this scenario, curettage might be the primary cause of migration. In summary, the mechanism of IUD migration in this patient may differ entirely from previously reported cases where the IUD first migrated to the bladder before perforating into the urethral orifice (7).

The position of the intrauterine device (IUD) can be confirmed through ultrasound, abdominal X-ray, endoscopy, or CT scan. Generally, ultrasound and abdominal X-ray are considered the first choice methods for diagnosing IUD displacement due to their simplicity, convenience, and low cost. Transvaginal ultrasound (TVUS) provides clearer images than transabdominal ultrasound (TAUS) and is therefore a better option (6). Assessing the position of an extra-pelvic IUD using ultrasound alone can be challenging, while an abdominal X-ray can help detect IUDs that have migrated outside the pelvis. CT can further confirm the diagnosis and assess the relationship between the uterine IUD and surrounding organs. In this case, the patient’s IUD was located relatively superficially. Besides using TAUS, we also performed a perineal color Doppler ultrasound, obtaining clear positional images. The IUD may sometimes be mistaken for bladder stones. Cystoscopy can identify foreign bodies, possibly allowing for direct removal with foreign body forceps and checking for any bladder fistulas (6). Based on the patient’s history of a lost IUD, combined with abdominal and perineal ultrasound and pelvic X-ray results, we initially were confident in diagnosing the IUD as having migrated to the bladder. However, the correct diagnosis was only confirmed during cystoscopy. It has been proven that CT is an indispensable option when the specific position of the IUD displacement cannot be fully confirmed. It is worth noting that due to the difficulty of diagnosing the presence of the IUD during pregnancy and the lack of awareness of such cases among local doctors, comprehensive and careful examinations were not conducted, and appropriate follow-up after curettage was lacking.

Since no standard treatment protocol is available, we referred to the treatment methods for IUD migration into the bladder or urethral orifice. Cystoscopy can remove IUDs that have completely migrated into the bladder or are accompanied by small stone formation. For larger stones or IUDs partially penetrating the bladder, laparoscopic or open surgery is recommended (14, 15). However, there are reports of successfully removing partially migrated IUDs using cystoscopy or nephroscopy, though there is a risk of IUD breakage or conversion to laparoscopic surgery (7, 16–18). The surgical approach for treating migrated IUDs must be chosen carefully. After careful consideration, we opted for cystoscopic removal of the IUD partially migrated to the posterior urethra, and no IUD breakage was observed during the procedure. We believe that endoscopic surgery is the preferred method for handling IUD migration. If unsuccessful, laparoscopic or open surgery can be pursued.

Despite the risks, removing the IUD during pregnancy is necessary for patients who choose to continue their pregnancy (19). The literature mentions three primary techniques: hysteroscopic IUD removal, ultrasound-guided hysteroscopic IUD removal, and ultrasound-guided forceps IUD removal (20). Given the condition’s rarity, no single technique has been established as superior. Based on the experience of a single center, Guglielmo Stabile et al. provided some tips: use a 3 to 5 mm hysteroscope to avoid cervical dilation, maintain low infusion pressure during the procedure to prevent potential damage to the gestational sac and displacement of IUD fragments, and heat the distension medium to 30°C to reduce vasoconstriction (21). It is worth noting that the surgical approach should be chosen based on the actual situation due to differences in equipment and expertise across centers.

The patient reported a positive experience with the treatment for the IUD migration. Initially anxious about the diagnosis, she appreciated the clear explanations and reassurance from the medical team. The cystoscopic intervention under local anesthesia was tolerable with minimal discomfort. Postoperatively, she was pleased with the absence of significant pain or complications, and the prompt removal of the catheter after ten days allowed her to resume normal activities. She expressed satisfaction with the resolution of her symptoms and was relieved by the lack of long-term urinary or vaginal issues.

## Conclusion

IUD is a simple, safe, effective, and reversible contraceptive method, but IUD migration is a rare yet serious complication. For patients with a history of IUD placement, particularly when the IUD has not been removed, curettage must be performed with great caution. Follow-up with pelvic X-rays or ultrasound examinations is necessary. Follow-up with pelvic X-rays or ultrasound examinations is essential. The posterior urethra is a very rare site for migration, and it may not present with significant LUTS symptoms or recurrent urinary tract infections. Gynecologists or urologists should carefully differentiate the diagnosis and not be limited to bladder migration. The mechanism of posterior urethral migration is not clear. Uterine perforation may occur early after IUD insertion, with subsequent urethral migration influenced by curettage or other factors. Endoscopic removal of the IUD should be the first-choice treatment for urethral migration, with laparoscopic or open surgery as necessary alternatives.

## Data Availability

The original contributions presented in the study are included in the article/supplementary material, further inquiries can be directed to the corresponding author.
